# Wenshen Zhuanggu formula inhibits tumor-exosomes induced bone pre-metastasis niche formation in primary breast cancer mice

**DOI:** 10.1186/s13020-025-01136-8

**Published:** 2025-06-16

**Authors:** Qionglian Huang, Hanjuan Ning, Jue Wang, Weiwei Liu, Sheng Liu, Jianyi Wang, Xianghui Han

**Affiliations:** 1 Institute of Chinese Traditional Surgery, Longhua Hospital, Shanghai University of Traditional Chinese Medicine, Shanghai, 200032 China; 2 Department of Liver Disease, Shanghai Yueyang Integrated Traditional Chinese Medicine and Western Medicine Hospital Affiliated to Shanghai University of Traditional Chinese Medicine, Shanghai, 200437 China

**Keywords:** Breast cancer, Bone pre-metastasis niche, Exosome, Wenshen Zhuanggu formula, Bone microenvironment

## Abstract

**Background:**

The therapy for breast cancer bone metastasis (BCBM) still needs profound investigation. Focusing on the formation of bone pre-metastasis niche (BPMN) is a crucial tache for BCBM treatment. Wenshen Zhuanggu Formula (WSZG) is a typical traditional Chinese medicine (TCM) prescription with ability of clinically palliating bone pain and ameliorating the quality of life of BCBM patients. This study aims to elucidate the inhibitory effect and underlying mechanism of WSZG on BPMN formation induced by tumor-derived exosomes.

**Methods:**

Bone precursor cells were exposed to exosomes derived from MDA-MB-231BO cells (BO-exo, 50 μg/mL) and treated by WSZG (10 or 20 μg/mL) in vitro. A primary breast cancer mouse model with propensity of BPMN was established by pre-educating BO-exo and then subcutaneously injecting MDA-MB-231BO cells into mammary fat pads. After the modeled mice were orally administrated WSZG (6.5 or 13 g crude herb kg^−1^ d^−1^) for 28 days, bone architecture and bone parameters of tibia were analyzed using micro-CT. The absorbed exosomes by bone precursor cells were investigated using confocal microscopy. Osteoclastic and osteoblastic differentiation were examined by TRAP, ALP and qRT-PCR. The levels of protein related to bone metabolism and extracellular matrix (ECM) were detected by ELISA and IHC methods.

**Results:**

The results showed that BO-exo elicited the vicious bone microenvironment to form BPMN in bone-tropic primary BC mice. WSZG encouragingly targeted BO-exo to hinder osteoclastic differentiation (*ACP5*, *c-Fos*, *Ctsk*, *MMP9*, *NFATc1*), improve focal bone lesions (BMD, Tb.N, Tb.Th, Tb.Sp, Conn-Dens. and BV/TV), and downregulate the expression levels of bone metabolism markers (ICTP, BALP, TRACP-5b) and bone ECM proteins (bone sialoprotein, elastin, fibronectin, osteopontin, collagen I and vitronectin).

**Conclusions:**

This study denotes the enhanced effects of BO-exo and highly inhibitive possibility by WSZG treatment on BPMN formation, shaping bone metastatic landscape of BC and informing the treatment of early-stage BCBM in terms of TCM.

**Supplementary Information:**

The online version contains supplementary material available at 10.1186/s13020-025-01136-8.

## Introduction

New 2.3 million female breast cancer (BC) patients have been diagnosed in 2022, and the mortality rate ranked fourth globally among 36 cancer types [1]. Distant metastases susceptibly develop in advanced BC, and 65–75% of advanced BC patients are liable to bone metastasis due to the abundant microvascular and nutrition in bone, which particularly causes shorter lifespan of patients [2, 3]. To date, radioisotopes, osteoclast inhibitors, chemotherapy, systemic endocrine therapy and supportive interventions are predominantly subjected for breast cancer bone metastasis (BCBM) therapy. Nevertheless, these therapeutic schemes are also controversial due to the failure of prolonging the overall survival of BCBM patients and inevitably serious side effects [4]. Thereby, the detection and prevention are emphasized in early-stage bone metastasis. Notably, developing drugs capable of preventing definite BCBM guides a novel direction for BCBM therapy.

It is well known that primary cancer cells intrinsically orchestrate bone microenvironment before metastasis, which is conducive to the subsequent colonization, adaptation, and survival of tumor cells in bones. This resulted fertile bone microenvironment is designated as bone pre-metastasis niche (BPMN) formation [5]. BPMN is principally characterized by aberrant bone remodeling, rearranged extracellular matrix (ECM), focal osteolytic lesions and bone metabolism disorder [6, 7]. The imbalance communication between osteoclasts and osteoblasts induced by primary BC cells usually leads to pre-osteoclasts hyperactivation, enhanced bone resorption, and finally aberrant bone architecture changes [8, 9]. BPMN is also composed of remodeled ECM involved the elevation of bone ECM proteins (elastin, fibronectin and collagen I, etc.) [6, 7]. All above biological processes are caused by such a biological phenomenon that primary BC cells secret various pro-metastasis factors (*i.e.* lysyl oxidases, L-plastin, and CCL-2) to directly influence osteoclasts in host microenvironment, resulting in the formation of focal osteolytic lesions [6, 10]. These factors are either soluble or contained in exosomes derived from BC cells.

Exosome, with diameter ranging 30–200 nm, is a subbranch of extracellular vesicles commonly existing in all organisms [11]. They intrinsically carry a wide range of bio-information including microRNAs, lipids, proteins and metabolites, etc., acting as key players in refractory disease outgrowth like malignant tumors [11]. For instance, integrins, the members of transmembrane proteins, are compositional cargo of cancer cells-derived exosomes, which attributes to intercellular communication like endocytosis as well as determines organotropic metastases including bone metastasis [12, 13]. Distinctly, tumor-originated exosomes usually trigger the initiation of pre-metastasis niche by educating the resident cells, recruiting immune cells and altering the stromal components in host regions [14]. Indicatively, the intervention to tumor-originated exosome functions and inhibition of BPMN formation point to BCBM treatment.

In recent, emerging clinical and basic evidence have highlighted the preventive values of traditional Chinese medicine (TCM) in BC metastases [15, 16]. Several RCT trials also demonstrated significantly decreased cancerous organotropism due to the combination of TCM prescriptions with chemotherapy compared to the controls [17–19]. Wenshen Zhuanggu Formula (WSZG), a TCM prescription formed by Psoraleae Fructus, Cnidii Fructus and Aconiti Lateralis Radix Praeparata, has been clinically employed for postoperative adjunctive therapy of patients with BCBM for a decade. In a multicenter, randomized, double-blind, controlled, prospective clinical trial, the participation of WSZG evidently degraded radiotherapy toxicity, reduced the incidence of bone-related events and ameliorated quality of life of BCBM patients, exhibiting a charming role in adjunctively treating BCBM [20]. Preclinical studies illustrated that WSZG dramatically restricted the malignant biological behaviors of BC cells in vitro and BCBM progression in vivo, with mechanisms involved in CCL5/CCR5, TGF-β1/Smads and IL-17B/IL-17BR signaling pathways [21–23]. While the benefits of WSZG in impeding formation of BPMN remain largely unexplained.

In this study, we examined the effects of WSZG on the formation of BPMN initiated by exosomes detached from highly bone-metastatic MDA-MB-231BO cells (BO-exo) in vitro and in vivo. This article sheds light to the malignant role of BO-exo in BPMN formation and highlights the preventive value of WSZG in early stage of BCBM.

## Materials and methods

### Plant materials and WSZG extract preparation

WSZG, consisting of Psoraleae Fructus (named Bu-Gu-Zhi in TCM, obtained by drying the mature fruits of *Cullen corylifolium* (L.) Medik.), Cnidii Fructus (named She-Chuang-Zi in TCM, obtained by drying the fruits of *Cnidium monnieri* (L.) Cusson) and Aconiti Lateralis Radix Praeparata (named Fu-Zi in TCM, sliced product of the processed lateral or daughter root of *Aconitum carmichaelii* Debeaux), was provided by the Pharmacy Department of Longhua Hospital. The WSZG extract was prepared using a previously established extraction protocol [23]. In brief, the three herbs were presoaked in 55% ethanol (1:10, w/v) as 5:5:3 (w) for 4 h, and then refluxed for 1.5 h. Being filtered, the residues were refluxed additionally with 55% ethanol (1:8, w/v) for 1 h. Two filtered decoctions were collected and concentrated by evaporating the excess liquid. WSZG extract was obtained in a freeze-drying way at a yield of 12.32% (weight of dried extract/weight of raw herbs). The quality of this extract was monitored by liquid chromatography-tandem mass spectrometry. Supplementary Fig. S1 showed the chromatographic profile of components in WSZG extract. The contents of psoralen, osthole and benzoylmesaconine were 14.17 mg/g, 25.60 mg/g and 1.17 mg/g, respectively.

### Cell culture

Human highly bone-metastatic MDA-MB-231BO cells (a gift from Dr. Toshiyuki Yoneda, Texas State University, TX, USA) [23], MCF-7 cells, pre-osteoclast RAW 264.7 cells and pre-osteoblast MC3 T3-E1 cells, human bone marrow-derived mesenchymal stem cells (hBMSCs) were maintained in Dulbecco’s modified Eagle’s medium (DMEM) contained 10% fetal bovine serum (FBS, Gibco, Australia) at 37 °C with 5% CO_2_.

### Isolation and identification of exosomes

The exosomes were isolated from MDA-MB-231BO and MCF-7 cells according to the previously described [13, 24]. Briefly, the 48 h-conditioned media, having cultured BC cells and initially with 10% exosome-depleted FBS, were collected and centrifuged twice (500 ×*g* for 5 min at 4 and followed 2000 ×*g* for 30 min at 4 C, after both were carried out with discarding the precipitations). Then, the supernatant was passed through a 0.22 μm porous filter to further deplete the non-exosome materials and then co-cultured with 16% polyethylene glycol (PEG, dissolved in distilled water) overnight at 4 ℃. Next, the mixed solution containing PEG-bound exosomes were centrifuged at 10,000 ×*g* for 60 min at 4℃ to harvest the primary precipitations. After that, by discarding the supernatant and resuspending the precipitations, a centrifugation at 120,000 × *g* for 70 min at 4 ℃ was performed to purify exosomes (XPN-100/90/80, Beckman Coulter, USA). The concentration of exosomes was quantified using a BCA kit. The protein expression of exosome markers was presented by western blot analysis. The morphology of exosomes was characterized using a 120 kV Transmission Electron Microscopy (TEM, Talos L120c G27, Thermo Fisher Scientific, USA). The particle size distribution and concentration identification of exosomes were determined by Nanoparticle Tracking Analysis (NTA, ZetaView® PMX-110, Particle Metrix Co., Ltd, Germany) and analyzed by ZetaView *8.04.02* SP2.

### Western blot analysis

The lysed total proteins from exosomes or cells were firstly denatured, then electrophoresed on 10% sodium dodecyl sulfate–polyacrylamide gel electrophoresis (Invitrogen, Life Technologies, Grand Island, NY, USA) and transferred onto poly vinylidene fluoride (PVDF) membranes. Next, the PVDF with samples was blocked at room temperature for 1 h and incubated with primary antibodies overnight at 4℃ (ITGβ3, TSG101, CD63 and CD81 were diluted as 1:1000; ITGα3, ITGαV, ACP5 and Ctsk were diluted as 1:500). By washing with tris buffered saline tween, the membranes were incubated in HRP conjugated antibodies at room temperature for 1–2 h. The reaction bands were obtained through the ChemDocTM Imaging System (Bio-Rad Laboratories, Inc., USA), and analyzed by Image J *1.54* Software.

### Establishment of a primary BC mouse model and drug administration

Forty female nude mice (SPF degree, aging 6 weeks, 18.3 ± 0.8 g) were purchased from Shanghai SLAC Laboratory Animal Co., Ltd. All animals were housed in SPF conditions and exposed to humane care in accordance with the recommendations in the Guide for the Care and Use of Laboratory Animals of the National Institutes of Health. The protocols were approved by Longhua Hospital Affiliated to Shanghai University of TCM (Registration Number: LHERAW-24032). All mice (5 per group) were randomly divided into model, WSZG (6.5 or 13 g/kg WSZG), zoledronic acid (Zole), BO-exo, two BO-exo + WSZG and BO-exo + Zole groups according to weights. Based upon the previously described methods [6, 25], the mice were firstly pre-educated by exosomes before modeling. Briefly, 50 μg of exosomes were suspended in 100 μL PBS and intravenously injected into the nude mice every other day for 21 days. The same volume of PBS was also injected to the control groups. 5 × 10^5^ of luciferase-labeled MDA-MB-231BO cells suspended in 100 µL PBS (with 50% Matrigel) were injected directly into the fourth mammary fat pad of each mouse at the end of BO-exo education. Based on the selected dosages of drugs in our previous study [21], two doses of WSZG (*i.g.*, 6.5 and 13 g/kg, once a day), Zole (a positive control drug, *i.p.*, 0.6 mg/kg, twice a week) or same volume of normal saline (*i.g.*) were administrated to the primary BC mice on the second day after modeling for consecutive 28 days, respectively. At the endpoint of drug treatment, the mice were humanely euthanized with 1% pentobarbital sodium solution. The blood, tumors and bones of BC mice were harvested. Tumor volumes in different groups were measured and calculated using the formula (length × width^2^)/2 [25].

### Bioluminescence imaging (BLI)

BLI was performed to confirm injection and monitor tumor growth progression every 7 days using an Xenogen IVIS 200 Imaging System (Caliper Life Sciences) upon injection of D-luciferin (40901ES01, Yeasen Biotechnology (Shanghai) Co., Ltd.).

### Micro-computed tomography (micro-CT) analysis

Micro-CT analysis was conducted to elucidate the information related to osteolytic lesions in tumor mice. The harvested tibia samples were firstly fixed with 4% paraformaldehyde for 24 h, and then scanned for creating three-dimensional images through the μCT 100 micro-CT scanner (SCANCO, Switzerland). Quantitative morphometric analyses of trabecular bone were performed by selecting a region of interest 0.5 mm below the tibial growth plate.

### Enzyme linked immunosorbent assay (ELISA)

According to the instruction of manufacturer’s specification, ELISA was performed to detect the serum levels of tartrate-resistant acid phosphatase 5b (TRACP-5b), bone alkaline phosphatase (BALP) and cross-linked carboxyterminal telopeptide of type I collagen (ICTP) in tumor mice from different groups.

### Quantitative real-time PCR (qRT-PCR)

Total RNA was acquired from RAW 264.7, MC3 T3-E1 cells or bone tissues applying a TRIzol reagent (Invitrogen, USA). The primary RNA concentration was measured using an ultra-micro-spectrophotometer (DS-11, DeNovix Inc., USA) and reverse-transcribed into cDNA following the concomitant instructions. The sequent samples with SYBR Green Master Mix (Thermo Fisher Scientific, Japan) were subjected for the performance of qRT-PCR employing an initial cycle (95 ℃ for 60 s), followed by up to 40 cycles, of each with 95 ℃ for 15 s, 60℃ for 15 s, and 72 ℃ for 45 s by the QuantStudio 3 real-time PCR system (Applied Biosystems, USA). The fold changes in relative mRNA expression were measured through the standard 2^−ΔΔCt^ method. Table 1 lists the primer sequences of the target genes.

**Table 1:** Sequences of forward and reverse primers applied in qRT-PCR analysis

Genes	Sequences
mouse ACP5	Forward: CACTCCCACCCTGAGATTTGT
	Reverse: CATCGTCTGCACGGTTCTG
mouse ALP	Forward: ATAACGAGATGCCACCAGAGG
	Reverse: TTCCACATCAGTTCTGTTCTTCG
mouse c-Fos	Forward: GGGTTTCAACGCCGACTAC
	Reverse: CTGTCACCGTGGGGATAAAG
mouse Ctsk	Forward: GGTGGTGGGCTATGGCAC
	Reverse: GCAGGCGTTGTTCTTATTCC
mouse MMP9	Forward: CCTGTGTGTTCCCGTTCATC
	Reverse: AACTACGGTCGCGTCCACTC
mouse NFATc1	Forward: CTCCCGTCACATTCTGGTCC
	Reverse: GGCTGCCTTCCGTCTCATAG
mouse OCN	Forward: AGGAGGGCAATAAGGTAGTGAAC
	Reverse: AGGCGGTCTTCAAGCCATAC
mouse Osx	Forward: ATGGCGTCCTCTCTGCTTG
	Reverse: TGAAAGGTCAGCGTATGGCTT
mouse Runx2	Forward: CTACCCAGCCACCTTTACCTAC
	Reverse: GAACTGATAGGATGCTGACGAAG

### Exosome uptake assay

RAW 264.7 and hBMSC cells are key bone precursor cells in studying osteogenesis and osteoclastic differentiation [26, 27], which dictates the bone homeostasis. Hence, BO-exo (5, 10, 25, 50 and 100 μg/mL) were labelled with PKH26 (a red exosomal label fluorescent dye, Umibio, China) and added into RAW 264.7 cells or hBMSCs for 16 h. Confocal microscopy was used for investigating the absorbed exosomes by these two cells. Additionally, the comparison of the uptake ability to BO-exo and MCF-7-exo by the two bone precursor cells was carried out. Lastly, RAW 264.7 cells and hBMSCs were grouped into control, WSZG (10 and 20 μg/mL), BO-exo (50 μg/mL) and two BO-exo + WSZG, and then seeded in confocal dishes overnight. The cells were co-cultured with BO-exo and treated by WSZG extract for 16 h and fixed in 4% paraformaldehyde at room temperature for 5 min. Images were captured employing the confocal laser scanning microscopy (Zeiss LSM-800, Oberkochen, Germany).

### Osteoclast differentiation

For osteoclast induction, RAW 264.7 cells were seeded in 24-well plates at a concentration of 8 × 10^3^ cells/well, and cultured overnight. Then, two drug-treated schemes were performed. (1) WSZG (10 or 20 μg/mL) and 50 μg/mL BO-exo were designed to treat RAW 264.7 cells for 24 h. (2) MDA-MB-231BO cells were pretreated with 10 or 20 μg/mL of WSZG for 24 h and BO-exo were isolated. Then RAW 264.7 cells were cultured with conditioned media containing BO-exo (50 μg/mL) for 24 h. Next, old media were removed, and fresh complete culture media supplemented with 50 ng/mL recombinant murine ligand (RANKL, PeproTech, USA) and 30 ng/mL macrophage colony-stimulating factor (M-CSF, HY-P70553, MCE, China) were added into the plates for 4 consecutive days. By discarding the old media and washing the cells for three times, the cells were fixed with 4% paraformaldehyde. Based upon these, tartrate resistant acid phosphatase (TRAP) assay was performed according to the manufacturer’s specification (G1492, Solarbio, China). TRAP positive rates were measured by Image J *1.54* Software.

### Osteoblast differentiation

For osteoblast induction, MC3 T3-E1 cells were seeded in 24-well plates at a concentration of 1 × 10^4^ cells/well. With the treatment of WSZG and exosomes for 24 h (the same concentration with osteoclast differentiation), osteogenic differentiation medium (PD-003, Procell, China) was added into the plates for 7 consecutive days, and the medium was replaced every 2 days. Based upon these, the activity of alkaline phosphatase (ALP) was measured using an ALP kit (P0321S, Beyotime, China) according to the manufacturer’s specification. Meanwhile, ALP staining was conducted using a pluripotent stem cell ALP color development kit (C3250S, Beyotime, China).

### Immunohistochemistry (IHC), ALP and TRAP staining on bone tissues

Initially fixed by 4% paraformaldehyde for 2 days, the tibia tissues were decalcified in regularly changed 14% EDTA for 28 days. Samples were then embedded in paraffin, and 5 μm sections were gained. The sections were dehydrated using xylene and ethanol series for IHC, ALP and TRAP analyses.

As for IHC analysis, sodium citrate buffer was used for antigen retrieval and 5% BSA was chosen for blocking (30 min). The blocked sections were incubated with indicated primary antibodies overnight at 4 °C. All antibodies including bone sialoprotein (BSP), elastin, fibronectin, osteopontin (OPN), collagen I and vitronectin were diluted as 1:50. After incubating with HRP-conjugated anti-rabbit secondary antibody (A0352, Beyotime, China, 1:50) at room temperature for 60 min, the slices were then rinsed by PBS for 3 times. SABC kit (P0603, Beyotime, China) and DAB (P0203, Beyotime, China) were successively applied to incubate the slices at room temperature for 0.5 h, both of which were subsequently followed a rinsing. The slices were then dehydrated through a series of ascending concentrations of ethanol and xylene, and covered with coverslips. The positive area of target proteins was measured by Image J *1.54* Software.

### Statistical analysis

All data were analyzed using one ANOVA analysis of variance with Bonferroni’s or Dunnett’s corrections for multiple comparisons as appropriate with a utility of SPSS *26.0* Software. All data are shown as mean ± standard error of mean (mean ± SEM). A value of *p* < 0.05 is considered as statistically significant.

## Results

### Identification of exosomes derived from MDA-MB-231BO and MCF-7 cells

**Fig. 1:**
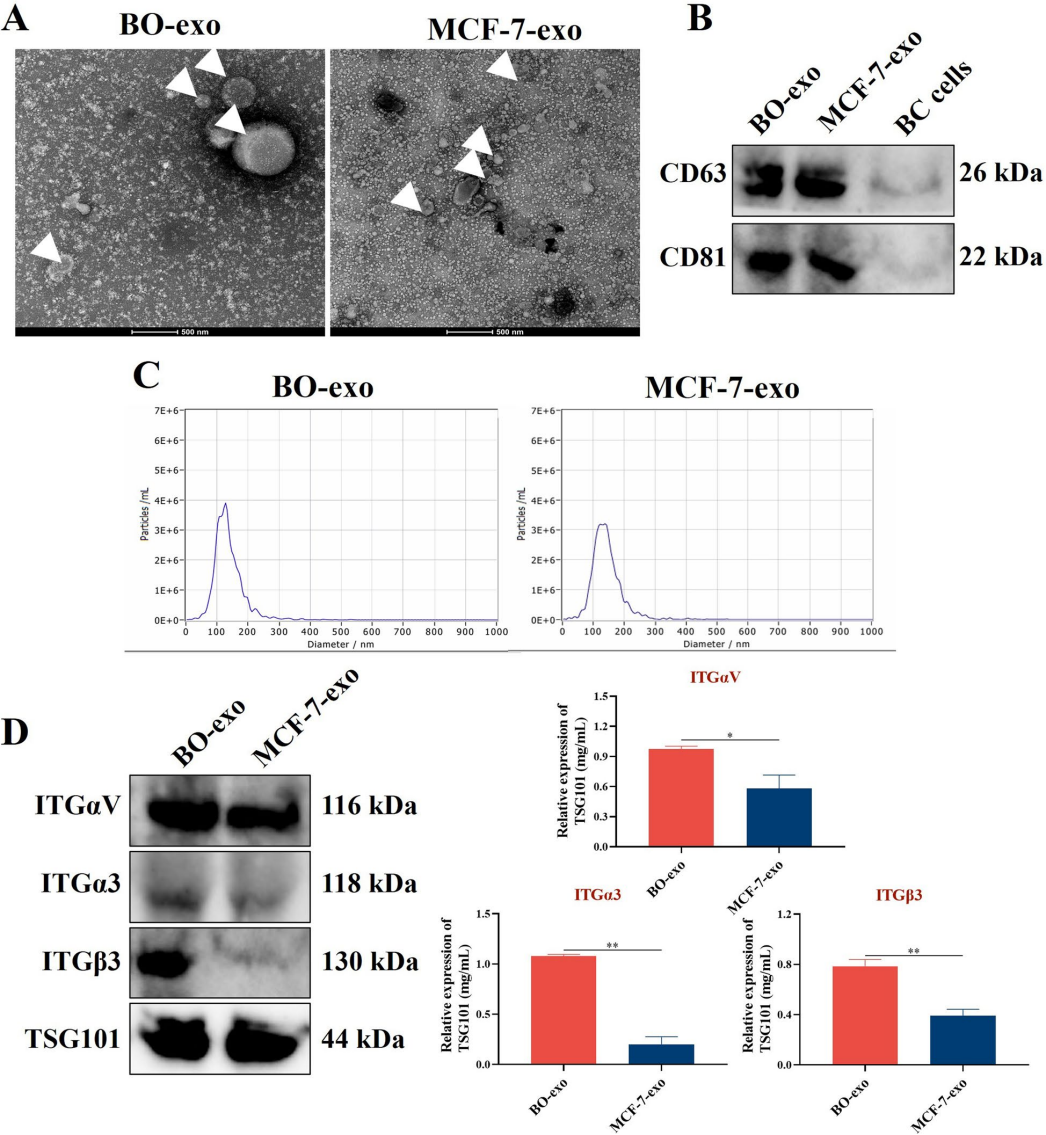
Identification of exosomes derived from MDA-MB-231BO and MCF-7 cells.** A** Representative transmission electron microscopy images of exosomes for morphology identification, (Scale bars = 500 nm). **B** Western blot analyses of exosome markers CD63 and CD81 validation. **C** Nanoparticle tracking analyses of exosomes for concentration confirmation and particle size distribution. **D** Western blotting analyses of ITGαV, ITGα3 and ITGβ3 in exosomes isolated from MDA-MB-231BO (BO-exo) or MCF-7 (MCF-7-exo) cells. All data are presented as means ± SEM (*n* = 3). *p < 0.05, **p < 0.01. All data are analyzed at least three independent experiments. *ns* no significant difference

Exosomes were isolated from the supernatant of MDA-MB-231BO and MCF-7 cells using a PEG-participation method [13, 24]. The morphology of the extracted exosomes was round or oval in shape under TEM observation (Fig. 1A). The positive expression of exosome marker proteins including CD63 and CD81 was validated in exosomes from the two BC cells by western blotting (Fig. 1B). What’s more, NTA showed that the concentration of exosomes from MDA-MB-231BO and MCF-7 cells was 3.1 × 10^11^ and 1.7 × 10^12^ particles/mL, respectively. And the percentage of isolated particles with diameter less than 130 nm was 97.4% and 97.3%, indicating the exceptional advantage of high yield by applying PEG-based precipitation methodology to obtain exosomes (Fig. 1C). These results above are consistent with the typical physical or biological characteristics of exosomes. Furthermore, the integrins carried by exosomes have been demonstrated crucial in bone metastasis progression [12, 28]. Therefore, the protein expression of ITGβ3, ITGα3 and ITGαV in exosomes from the two BC cells were analyzed. In result, the protein levels of these integrins were observed higher in bone-tropic BO-exo than free-bone-tropic MCF-7-exo (Fig. 1D, *p* < 0.05 or *p* < 0.01).

### WSZG inhibited the uptake abilities of pre-osteoclastic and pre-osteoblastic cells to BO-exo

**Fig. 2:**
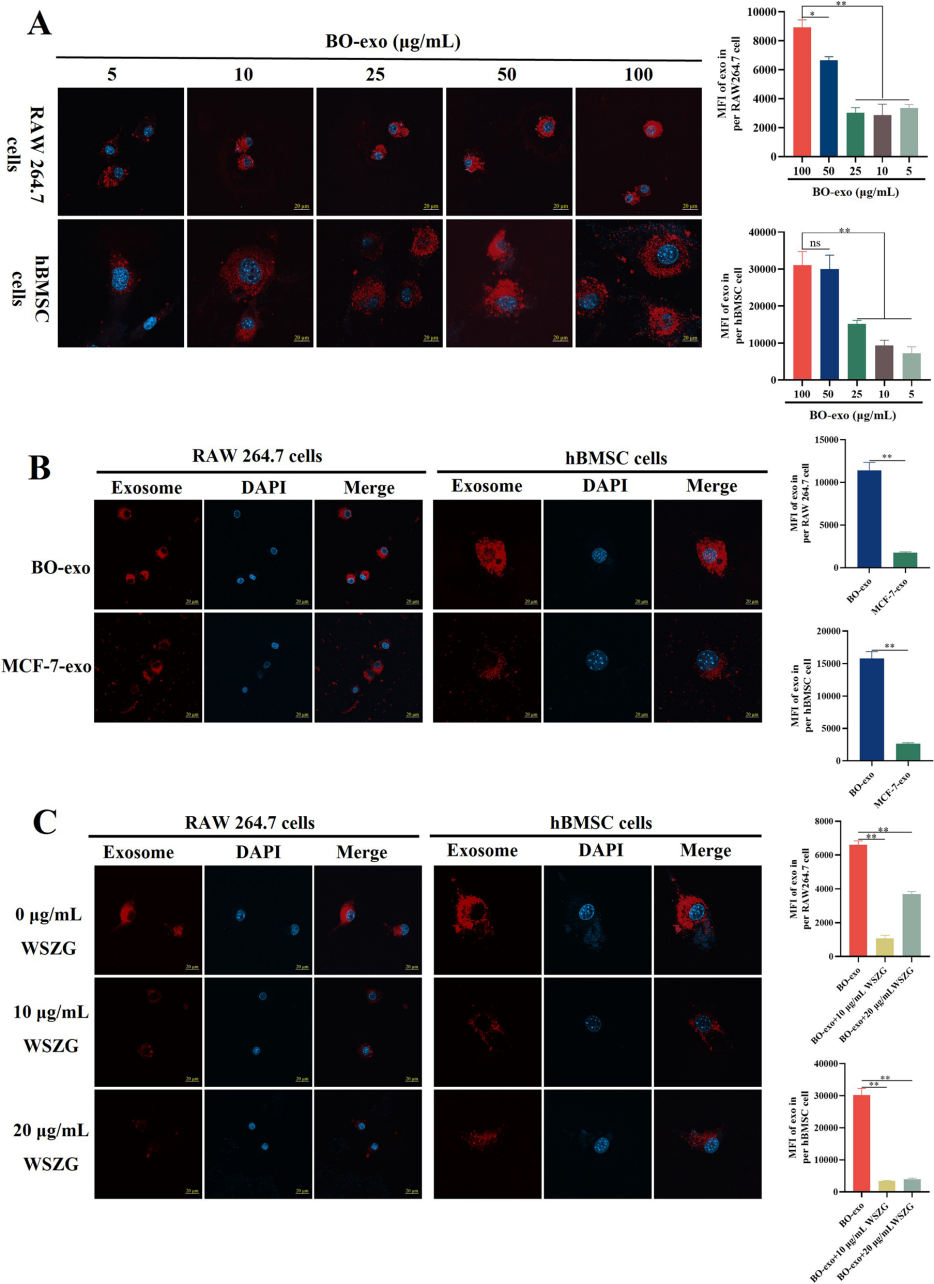
WSZG inhibited the uptake abilities of pre-osteoclastic and pre-osteoblastic cells to BO-exo. **A** Representative images and quantitative analysis of BO-exo (5, 10, 25, 50 and 100 μg/mL) taken up by pre-osteoclastic (RAW 264.7) and pre-osteoblastic (hBMSC) cells for confirmation of optimal exosomal concentration. **B** The comparison to the uptake abilities of RAW 264.7 or hBMSC cells to 50 μg/mL BO-exo and MCF-7-exo. **C** Repressive uptake by RAW 264.7 or hBMSC cells to 50 μg/mL BO-exo after 10 or 20 μg/mL WSZG treatment. Scale bars = 20 μm. All data are presented as means ± SEM. *p < 0.05, **p < 0.01. All data are analyzed at least three independent experiments. *ns* no significant difference

MDA-MB-231BO cells, with a propensity of high bone metastasis, are the subbranch of MDA-MB-231 TNBC cell lines [21]. The uptake of bone precursor cells to exosomes is one prerequisite of cancerous pre-metastasis niche formation [6]. We firstly determined the most suitable concentration of BO-exo taken up by RAW 264.7 cells or hBMSCs. Hence, 5, 10, 25, 50 and 100 μg/mL of BO-exo were co-cultured with the two precursor cells, and the confocal microscopy was designed to confirm the MFI of each group. Though the red fluorescent granular within each precursor cell increased gradually with the elevation of concentration (quantified by MFI of each cell), the MFI in 50 and 100 μg/mL of BO-exo groups exhibited a distinct increase compared to other groups (Fig. 2A). In consequence, 50 μg/mL was chosen as the optimal concentration of exosomes for the subsequent analyses.

We proceeded to compare the different uptake capabilities of the two precursor cells to bone-tropic (BO-exo) and free-bone-tropic exosomes (MCF-7-exo). The same concentration exosomes were stained with PKH26 and then co-cultured with RAW 264.7 cells or hBMSCs for 16 h. In result, significantly higher density of MFI was observed in BO-exo groups than that in MCF-7-exo groups, suggesting that the two precursor cells were preferable to take up BO-exo rather than MCF-7-exo (Fig. 2B, *p* < 0.01). More compellingly, visualized red fluorescent granular in cytoplasm was markedly mitigated through the treatment of 10 or 20 μg/mL WSZG in comparison with the control groups (Fig. 2C, *p* < 0.01). Taken together, these results imply that WSZG is capable of degrading the uptake abilities of pre-osteoclast and pre-osteoblast cells to BO-exo.

### WSZG inhibited the BO-exo-induced osteoclast and osteoblast differentiation

**Fig. 3:**
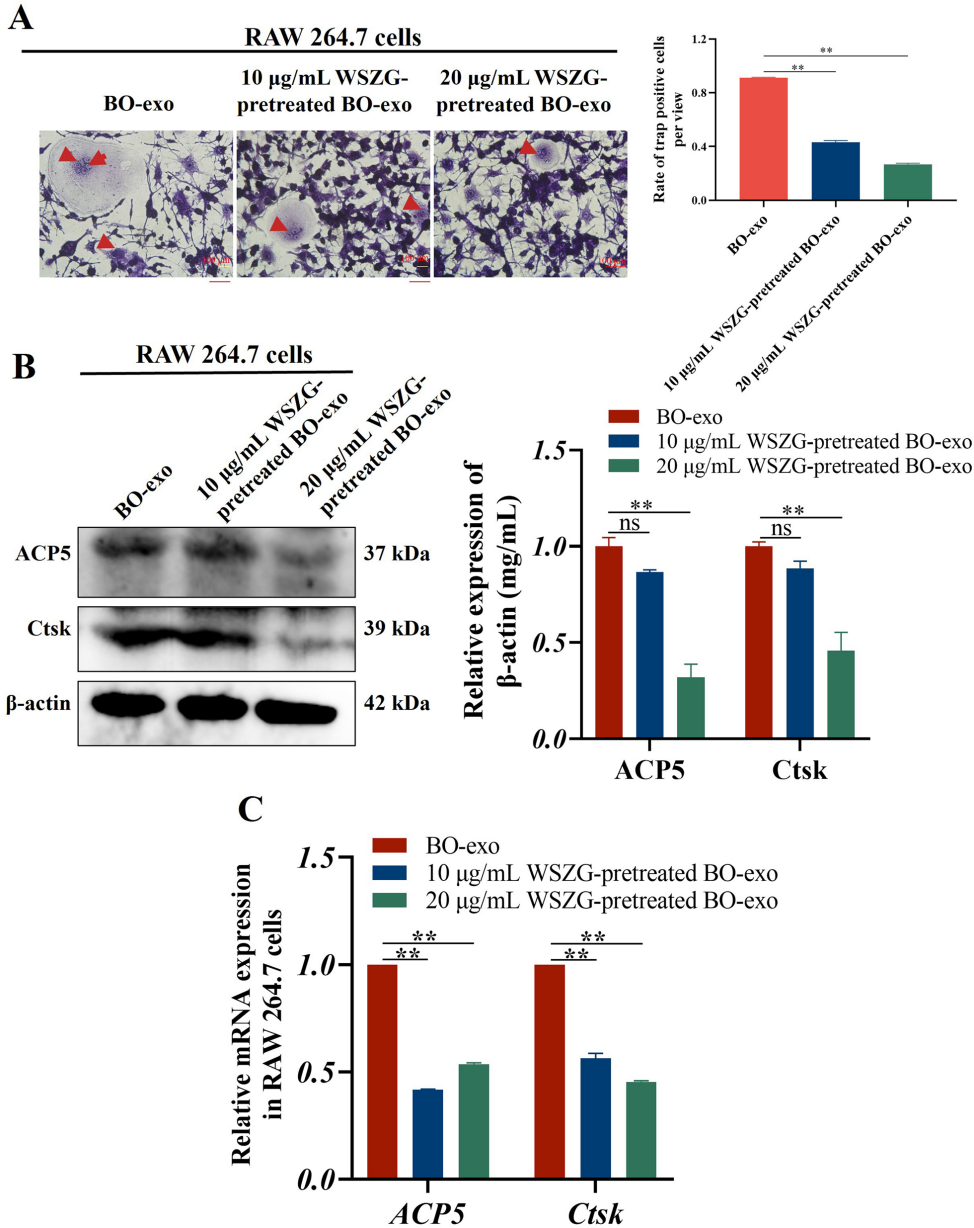
WSZG inhibited the BO-exo-induced osteoclast and osteoblast differentiation. All cells were treated by 10 or 20 μg/mL WSZG and exposed to 50 μg/mL BO-exo. **A** Representative images and quantitative analysis of TRAP staining of RAW 264.7 cells after 4 consecutive days for osteoclastic differentiation. **B** mRNA levels of osteoclastic differentiation markers (*c-Fos*, *ACP5*, *NFATc1*, *Ctsk*, *MMP9*) in RAW 264.7 cells detected by qRT-PCR analysis. Representative images of ALP staining (**C**) and ALP activity (**D**) in MC3 T3-E1 cells after 7 consecutive days for osteoblastic differentiation. **E** mRNA levels of osteoblastic differentiation markers (*ALP*, *OCN*, *Osx*, *Runx2*) in MC3 T3-E1 cells detected by qRT-PCR analysis. Scale bars = 100 μm. All data are presented as means ± SEM. *p < 0.05, **p < 0.01. All data are analyzed at least three independent experiments. *ns* no significant difference

We performed TRAP, ALP staining and qRT-PCR assay to illustrate osteoclast, osteoblast differentiation and the expressional levels of related markers, respectively. TRAP staining exhibited that the exposure of BO-exo to RAW 264.7 cells was able to remarkably rise the number of osteoclasts (with properties of larger and multi-nucleus [28, 29]) compared to the control group (*p* < 0.01), indicating that BO-exo has a potential to accelerate the differentiation of osteoclasts and enhance osteoclastic bone resorption. Interestingly, we found that the treatment of 10 or 20 μg/mL WSZG to RAW 264.7 cells distinctly attenuated the differentiative process of osteoclasts based on BO-exo exposure (Fig. 3A, *p* < 0.01). Histological evidence showed that BPMN, related to BC, characterized enhanced osteoclastic bone resorption and attenuated osteoblastic bone formation [30]. The results of ALP staining displayed that the amounts of stained cells (bluish-purple) in BO-exo group were remarkably less than those in the control group (*p* < 0.05 or *p* < 0.01). What’s more, the treatment of WSZG obviously increased the amounts of bluish-purple cells exposed to BO-exo or not (Fig. 3C, *p* < 0.05 or *p* < 0.01). Consistent findings were also seen in ALP activity (Fig. 3D, *p* < 0.05 or *p* < 0.01). Collectively, these results predicted that the absorbed BO-exo distinctly boosted osteoclastic differentiation and slowed down the differentiation of MC3 T3-E1 cells into osteoblasts, while the treatment of WSZG was capable of reversing the above results.

The data of qRT-PCR acknowledged a dramatically enhanced efficacy of osteoclastic markers including *ACP5*, *c-Fos*, *Ctsk*, *MMP9*, *NFATc1* in differentiated RAW 264.7 cells that were co-cultured with BO-exo. Surprisingly, nearly normal mRNA expression levels of these markers in RAW 264.7 cells were validated by treating 20 μg/mL WSZG for 24 h (Fig. 3B, *p* < 0.05 or *p* < 0.01). Similar results also could be seen in the protein expression of osteoclastic markers in RAW 264.7 cells after WSZG treatment (Supplementary Fig. S2,* p* < 0.05 or *p* < 0.01). Conversely, WSZG has a strong potential in facilitating the BO-exo-inhibited mRNA expression of osteoblastic markers including *ALP*, *OCN*, *Osx*, *Runx2* in differentiated MC3 T3-E1 cells (Fig. 3E, *p* < 0.01). All results above shed light to that BO-exo disrupts the balance of osteoclast and osteoblast in terms of BPMN, which is probably rescued by WSZG.

To explore whether WSZG modifies BO-exo itself, we firstly pretreated MDA-MB-231BO cells with 10 or 20 μg/mL of WSZG and then the isolated BO-exo were exposed to RAW 264.7 cells. Similar to the results above, the rate of TRAP-positive cells was decreased by 52.51%−70.80% in WSZG-pretreated BO-exo groups compared to the untreated BO-exo groups (Supplementary Fig. S3 A, *p* < 0.01). Compared to BO-exo group, the mRNA and protein levels of two key osteoclastic marker Ctsk and ACP5 were significantly downregulated in WSZG-pretreated BO-exo groups (Supplementary Fig. S3B, C, *p* < 0.01). Taken together, these findings indicate that WSZG directly influences BO-exo, thus prohibiting the osteoclast differentiation owing to BO-exo. Additionally, the protein expression of ITGβ3 and ITGα3 were strongly decreased in WSZG-pretreated BO-exo groups relative to BO-exo group (Supplementary Fig. S4, *p* < 0.05 or *p* < 0.01).

### Influence of WSZG on BO-exo-enhanced tumor burden in primary BC mice

**Fig. 4:**
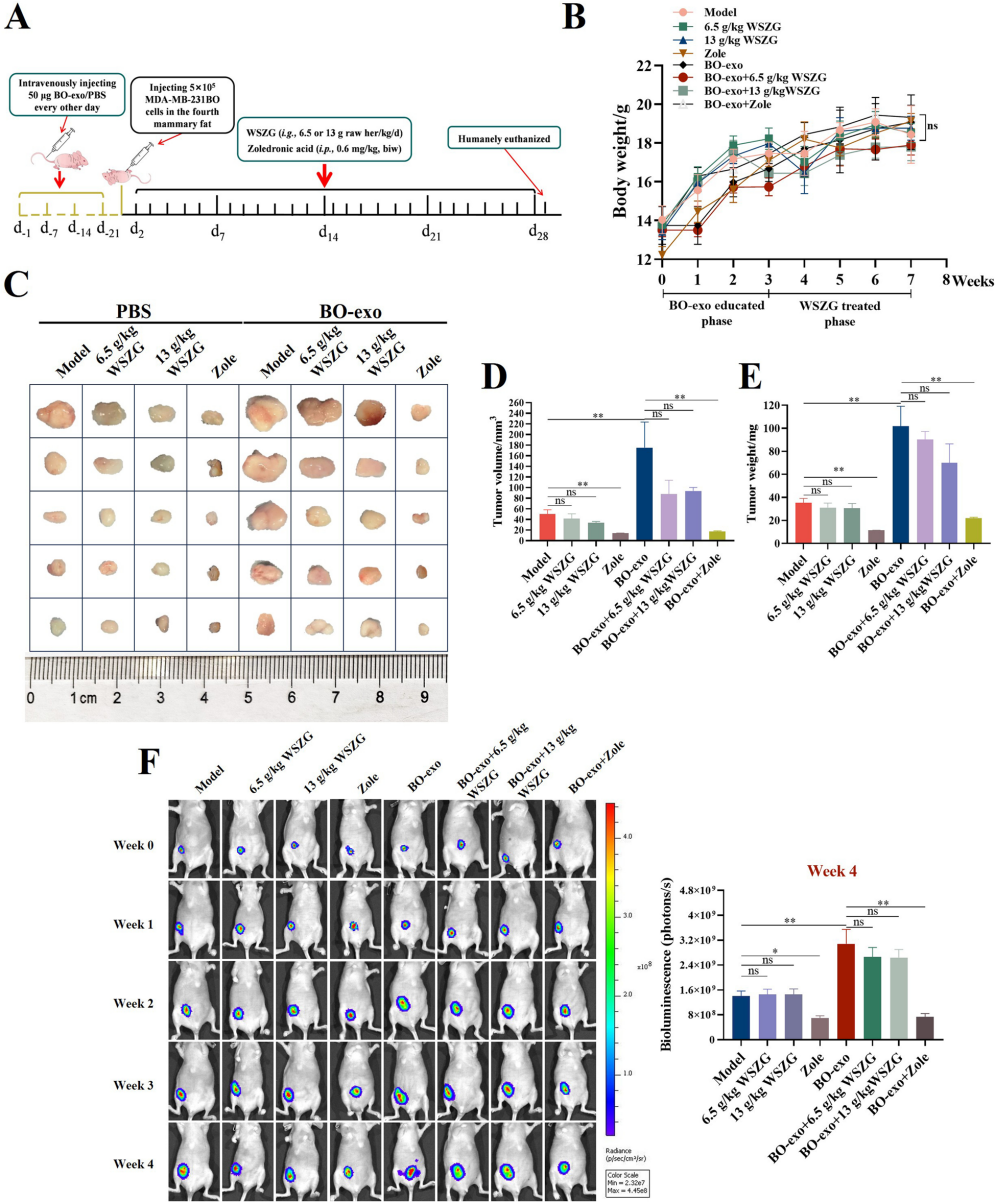
WSZG influenced the BO-exo-enhanced tumor burden in primary BC mice. **A** Schematic illustration of experimental processes involved in exosome education, establishment of a primary BC model, and drug treatment. **B** The body weight of mice was quantitated every 7 days. Representative photographs of xenografted tumor sizes and BLI images were exhibited in (**C**) or (**F**). Tumor volume (**D**) and tumor weight (**E**) of primary BC mice were quantitated at the end point of experiment. All data are presented as means ± SEM (*n* = 5). *p < 0.05, **p < 0.01. *ns* no significant difference

A growing body of publication findings supported that the exosomes derived from primary cancer cells initiated the cancer progression with a promotion of tumor burden [6, 10, 31]. According to the existed study [6], we firstly pre-educated the mice with 50 μg BO-exo. Then a primary BC mouse model with a predisposition to bone was established by subcutaneously injecting MD-MB-231BO cells, and the modeled mice were subsequently administrated WSZG or Zole for 28 days (Fig. 4A). In result, we found no obvious differences of body weight among all groups (Fig. 4B, *p* > 0.05). Similar to the previous findings [32], significant facilitation of tumor burden was observed in BO-exo group compared to free-BO-exo group (model group), verified by the results of tumor weight (175.07 ± 48.32 mg *vs.* 35.23 ± 3.84 mg), tumor volume (119.44 ± 24.09 mm^3^ *vs*. 50.06 ± 8.10 mm^3^) and BLI (3.08 × 10^9^ photons/s *vs.* 1.40 × 10^9^ photons/s, *p* < 0.05 or *p* < 0.01). However, it seems that WSZG failed to rescue the boosted primary tumor growth granted by MD-MB-231BO cells (Fig. 4C–F, *p* > 0.05).

### WSZG inhibited BO-exo-stimulated osteoclastogenesis in bone tissues of primary BC mice

**Fig. 5:**
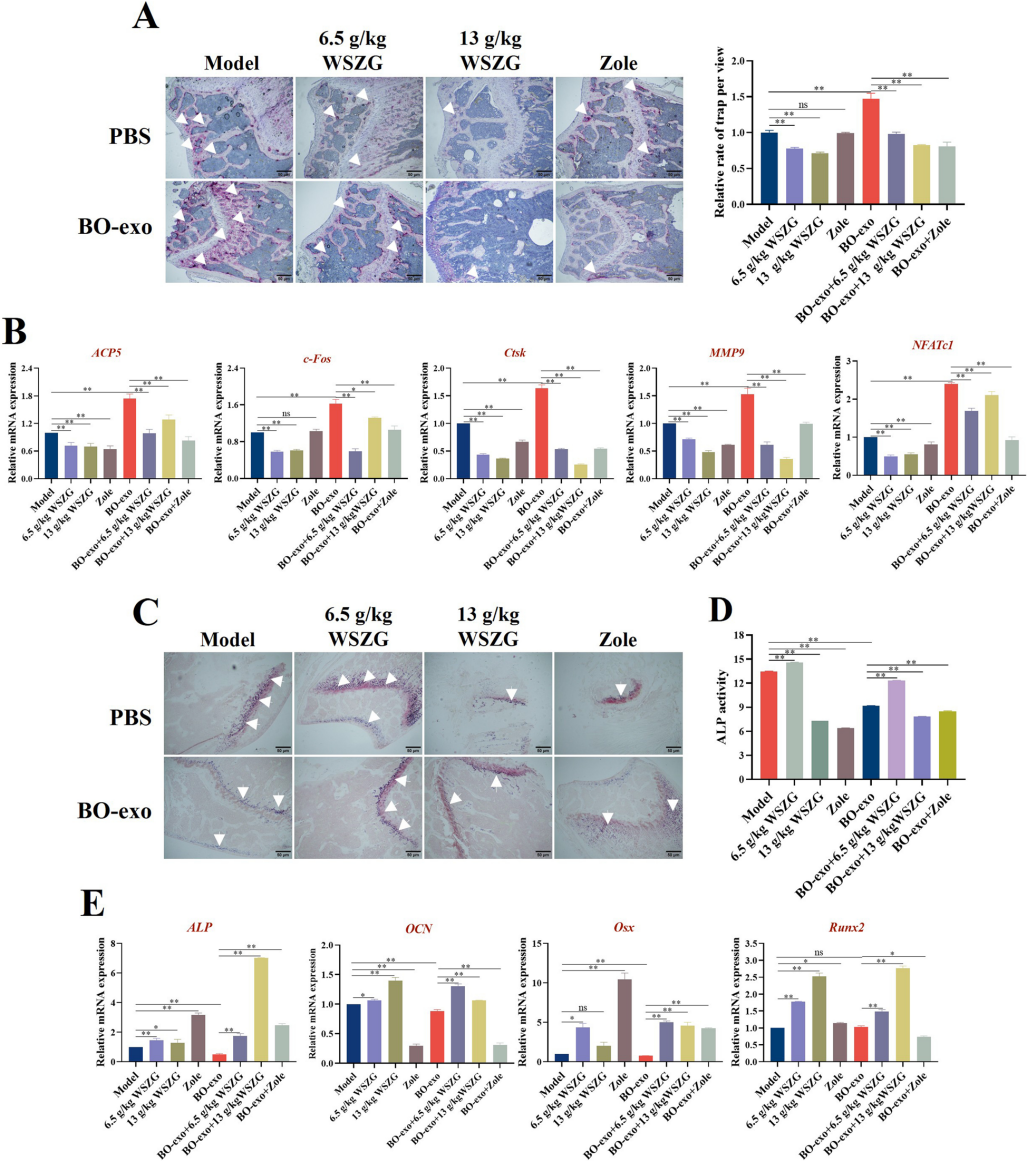
WSZG inhibited BO-exo-stimulated osteoclastogenesis in bone tissues of primary BC mice. **A** Representative images and quantitative analysis of TRAP staining in bone tissues after BO-exo education for 21 days and subsequent WSZG treatment for 28 days. The area with wine-red color was considered as TRAP^+^. Representative images of ALP staining (**C**) and ALP activity (**D**) in bone tissues. The area of bone baseline with bluish-purple were considered as ALP^+^. QRT-PCR analysis of osteoclastic differentiation (**B**) and osteoblastic differentiation (**E**) marker mRNA levels in bone tissues. Scale bars = 50 μm. All data are presented as means ± SEM (*n* = 3). *p < 0.05, **p < 0.01. *ns* no significant difference

The cancer-derived exosomes containing precise bioinformation usually set out for distant organs and launch the malignant metastasis [33–35]. In our results, BO-exo-educated mice exhibited remarkably elevated TRAP^+^ area compared to the model mice (without BO-exo education, *p* < 0.01), while the treatment of 6.5 or 13 g/kg WSZG exceptionally diminished TRAP^+^ area in the mice with or without BO-exo education (Fig. 5A, *p* < 0.01). Consistently, there was an exclusive elevation in mRNA levels of osteoclast differentiation markers (*ACP5*, *c-Fos*, *Ctsk*, *MMP9*, *NFATc1*) in BO-exo-educated mice compared to that in free-BO-exo mice (model group, *p* < 0.01). WSZG treatment was capable of strongly reducing the mRNA levels of related genes (*p* <0.05 or *p* < 0.01), and similar results were obtained in Zole groups (Fig. 5B, *p* < 0.05 or *p* < 0.01). These results indicate that WSZG is more sensitive to restrain BO-exo-educated osteoclast differentiation.

The imbalance of bone homeostasis was triggered by the strong activation of osteoclasts and abnormal osteoblast progression [9]. There was a clearly decreased ALP activity in BO-exo group by comparing to the model group (*p* < 0.01), while a significantly increased ALP activity was observed in 13 g/kg WSZG-treated groups compared to the model or BO-exo group (Fig. 5C, D, *p* < 0.01). QRT-PCR findings showed that the mRNA levels of several osteoblastic markers (*ALP*, *OCN* and *Osx*) in bone tissues were significantly lowered after BO-exo education (*p* < 0.01). Compared with the model and BO-exo groups, these osteoblastic gene levels were increased in WSZG-treated groups (*p* < 0.01). Zole treatment significantly enhanced ALP and Osx expression (Fig. 5E, *p* < 0.05 or *p* < 0.01). Taken together, the findings above implicate that the vicious disruption of bone homeostasis of BPMN is initiated by BO-exo, and WSZG reverses the situation.

### WSZG rescued the BO-exo-aggravated bone loss and focal osteolytic lesions in primary BC mice

**Fig. 6:**
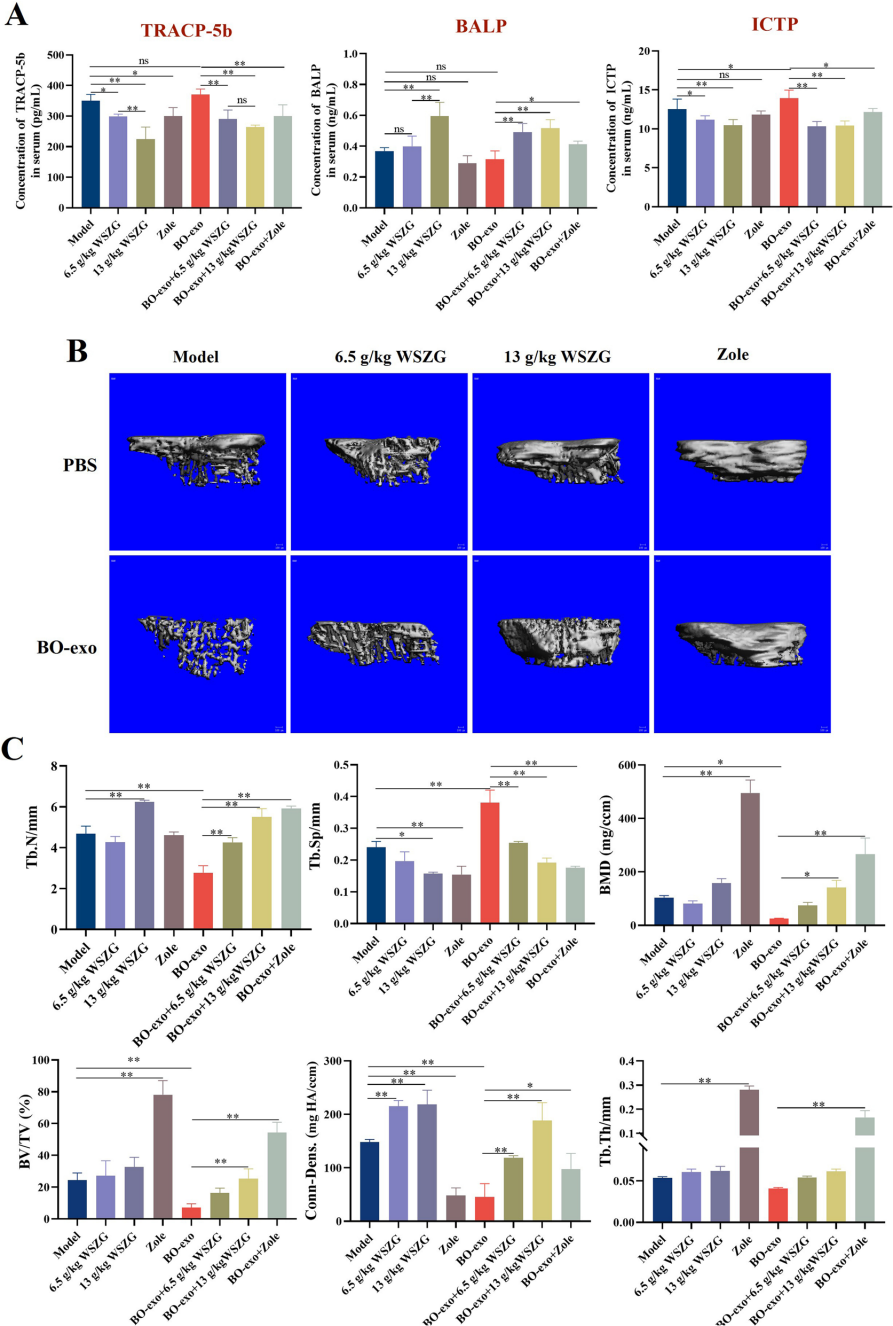
WSZG rescued the BO-exo-aggravated bone loss and focal osteolytic lesions in primary BC mice. **A** Serum levels of TRACP-5b, BALP and ICTP in tumor mice after BO-exo domestication and WSZG treatment by ELISA assay. **B** Representative images showing three-dimensional architecture after micro-CT reconstruction of the tibias from tumor mice. **C** Quantitative bone parameter analysis of the tibias from tumor mice in different groups, including Tb.N, Tb.Sp, BMD, BV/TV, Conn-Dens. and Tb.Th. Scale bar = 100 μm. All data are presented as means ± SEM (*n* = 3). *p < 0.05, **p < 0.01. *ns* no significant difference

ELISA assay was conducted to validate the levels of bone metabolism markers in serum of BC mice. We found that there was an obviously increased ICTP level in BO-exo group compared to the model group (without BO-exo-education, *p* < 0.05), which can be reduced by 6.5, 13 g/kg WSZG or 0.6 mg/kg Zole (*p* < 0.05 or *p* < 0.01). For BALP or TRACP-5b levels in serum, no significant changes were observed between the BO-exo and model groups, but WSZG or Zole treatment was able to elevate BALP level and reduce TRACP-5b level in case of BO-exo education (Fig. 6A, *p* < 0.05 or *p* < 0.01). All these results indicate that the pre-education of BO-exo to primary BC mice delicately boosted the osteoclast activities and suppressed the osteoblasts activities, while WSZG delayed the programmed processes in molecular aspect.

We performed micro-CT scans to detect the changes of bone mass and focal osteolytic lesion in tibia of primary BC mice (Fig. 6B). The bone microstructure of tibia in the model mice (without BO-exo-education) showed complete trabecular bone and focal osteolytic lesion in lower part of cortical bone. After BO-exo education, the cortical bone was seriously damaged with broken trabecular morphology and larger trabecular interval. Compared with the model or BO-exo group, the larger thickness trabecular bone and more intact cortical bone morphology displayed in WSZG-treated and Zole-treated groups, suggesting the encouraging ability of drugs to increase the area of cortical bone and protect the architecture of trabecular bone.

Bone parameters in tibia metaphysis were analyzed by micro-CT imaging system. Trabecular number (Tb.N), bone mineral density (BMD), trabecular thickness (Tb.Th), connectivity density (Conn-Dens.) and bone volume/tissue volume (BV/TV) strongly diminished, and trabecular space (Tb.Sp) evidently increased in BO-exo group compared to the model group (free-BO-exo education, *p* <0.05 or *p* < 0.01), which hints that osteoporosis was successfully developed in the primary BC mice by pre-education of exosomes. WSZG treatment dramatically improved these indexes in case of BO-exo education rather than in the absence of BO-exo education (Fig. 6C, *p* < 0.05 or *p* < 0.01), which implies higher susceptibility of WSZG to bone microenvironment under BO-exo education compared to without BO-exo education. Based on the gathered findings, BO-exo indeed aggravated the degree of osteoporosis while WSZG could protect the bone microstructure and strength of primary BC mice.

### WSZG inhibited BO-exo-elevated ECM protein expression in bone tissues of primary BC mice

**Fig. 7:**
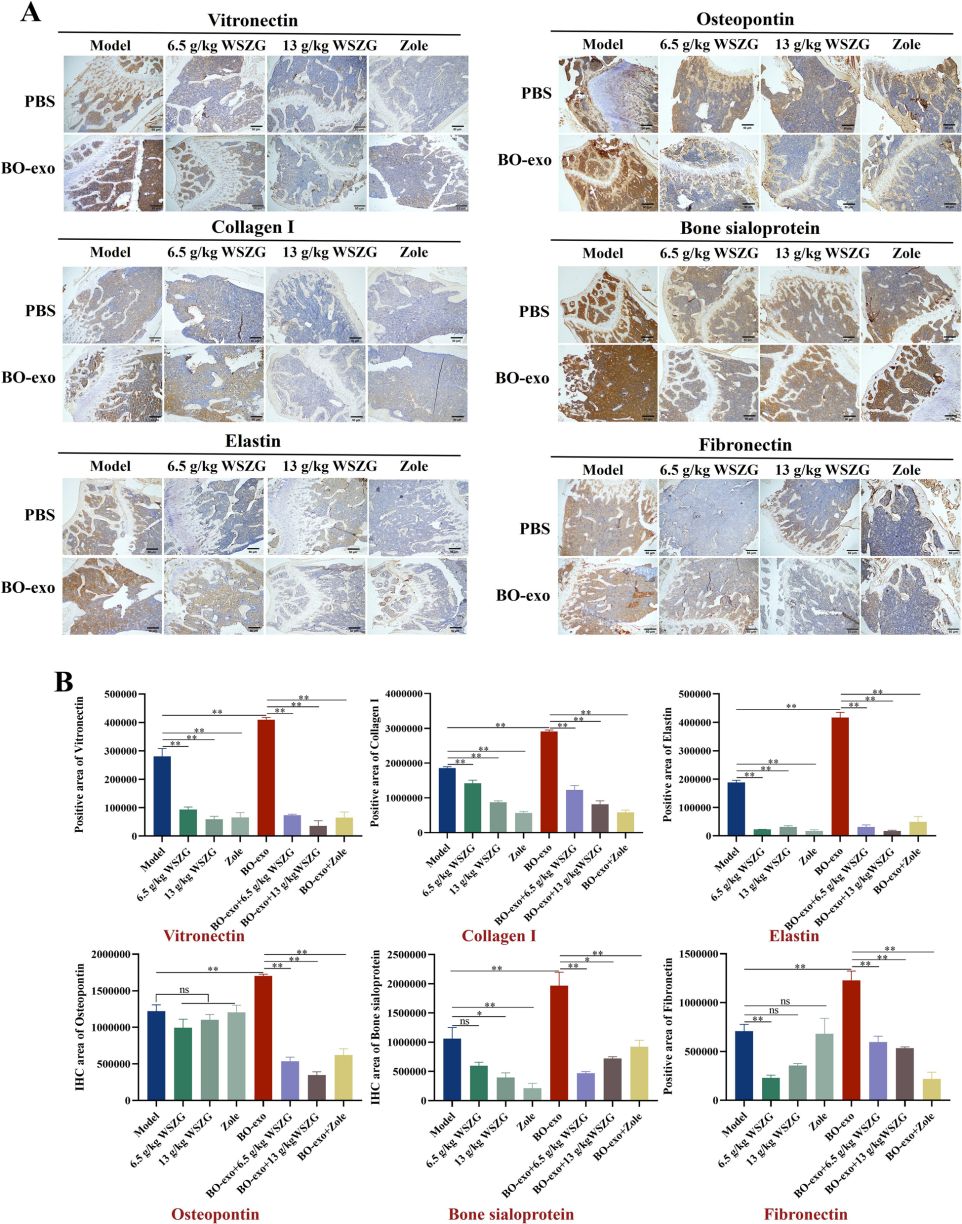
WSZG inhibited BO-exo-elevated ECM protein expression in bone tissues of primary BC mice.** A** Representative IHC images of bone ECM proteins including vitronectin, collagen I, elastin, osteopontin, bone sialoprotein, and fibronectin in different groups with or without BO-exo education and drug treatment. Scale bar = 50 μm. **B** Quantification analysis of bone ECM protein expression in tumor mice from different groups. All data are presented as means ± SEM (*n* = 3). *p < 0.05, **p < 0.01. *ns* no significant difference

ECM proteins are exclusively produced by multiple types of skeletal cells. Their aberrant expressions in bone herald the pathological alteration of bone microenvironment and incidence of osseous metastasis event [36]. With BO-exo education, there were remarkable elevation in IHC area of BSP, elastin, fibronectin, OPN, collagen I and vitronectin protein expression in bone tissues from BC mice (*p* < 0.01). And WSZG or Zole treatment led to more than half attenuation of these ECM protein expression (Fig. 7A, B, *p* < 0.05 or *p* < 0.01). Taken together, these data suggest that BO-exo strengthened the bone ECM rearrangement in primary BC mice while WSZG magically reversed them.

## Discussion

BCBM development consists of several stages. Firstly, primary BC cells recruit “seeds” to make bone more amenable for subsequent invasion to form BPMN. Secondly, metastatic BC cells set out to remote bone through bloodstream. Thirdly, these arrival BC cells induce dormancy or release a series of pro-metastasis factors to activate a cascade of metastasis-related events, initiating the vicious cycle. Ultimately, abundant BC cells proliferate in bone [37]. The formation of BPMN is a prerequisite for further evolution of BCBM [5], which involves osteoclast-osteoblast imbalance, ECM remodeling, focal osteolytic lesions and bone metabolism disorder [8, 9, 11]. Typically, the disruption of bone homeostasis is demonstrated as a paradigm of bone metastasis progression, which is caused by pro-metastasis factors including circulating tumor cells [38], exosomes [23], cytokines, chemokines and large oncosomes, etc. [39]. To our best knowledge, this is the first fundamental study on interception of exosome-induced BPMN by WSZG that is a conventional TCM formula applied in bone-metastatic clinical settings.

Exosomes are crucial players in establishing multi-organic pre-metastasis niche. The exosomes released by primary cancer cells are prone to recruit immune cells and affect metabolic reprogram of stromal cells to prompt the brain, lung, bone and liver pre-metastatic niche establishment [40–43]. Yuan *et al.* used exosomes from bone-tropism BC cells to established pre-metastasis microenvironment in bone tissues for 21 days, and found that the BPMN formation was correlated with enhanced differentiation of osteoclasts [6]. Furthermore, exosomal hsa-miR-940 dismissed by prostate cancer cells provoked the osteoblastic bone metastasis by interacting with the local mesenchymal cells [44], which involves in BPMN formation. Obviously, the bone microenvironment pre-educated by exosomes before the establishment of a primary cancer model has been subjected for imitating the formation of cancer-related pre-metastasis niche. To achieve this, we employed BO-exo derived from bone-tropism BC cells to treat bone parental cells in vitro and pre-educate bone microenvironment of primary BC mice, and investigated the preventive activities of WSZG in terms of BCBM progression.

New perspectives suggest that the uptake to tumor exosomes by bone marrow-derived macrophages in host regions perhaps predicts the initiation of pre-metastasis niche progression [6, 45]. This observation was conforming to our study, which demonstrated higher uptake rate (more red fluorescent granular presenting in cytoplasm) of pre-osteoclast and pre-osteoblast cells to exosomes originated from bone-tropic MDA-MB-231BO cells (BO-exo) rather than free-bone-tropic MCF-7 cells (MCF-7-exo). And recent studies have deduced that the preferential uptakes to exosomes by recipient cells are governed by several factors, one of which is the specific surface marker level on exosomes. For instance, the more CD47 or v-SNARE VAMP-3 on exosomes are positive correlation with higher exosome uptake by specific recipient cells [46, 47]. Additionally, the unique tumor exosomal integrins such as ITGα6β4 and ITGα6β1 may interact with cell-associated ECM, mediating exosome uptake in specific resident stromal cells in lung and liver tissues [12]. In this study, we confirmed higher integrin proteins including ITGβ3 and ITGα3 in bone-tropism exosomes. Based upon the findings above, we speculated that bone precursor cells (RAW 264.7 and hBMSC cells) specifically uptake bone-tropic tumor exosomes (BO-exo) to affect the progression of osteoclastic and osteoblastic differentiation with the help of ITGβ3 and ITGα3. What’s more, similar with the precious studies [8, 9, 11], BO-exo aggravated other typical traits of BPMN such as focal bone infrastructure destruction, bone loss, and bone ECM protein reprogram in primary BC mice. Collectively, our study proved distinct promotion of BPMN induced by BO-exo in vivo and in vitro.

In the present study, we validated the intervention of WSZG on bone-tropic exosomes (BO-exo) and found conducive efficacy of WSZG on inhibiting BO-exo-provoked BPMN formation through weakening the exosome uptake by bone precursor cells, thus halting osteoclastogenesis and bone resorption, rearranging bone ECM rigidity, attenuating bone loss and bone destruction. Interestingly, the education of bone-tropic exosomes (BO-exo) promotes larger tumor sizes in primary BC mice but WSZG treatment failed to reverse the state. We speculate that the therapeutic targets and bioactive components of WSZG predominantly focus on regulating bone microenvironment rather than primary tumor microenvironment in a concurrent body, which remains a matter of future studies. Emerging literatures have documented that the herbal drugs from WSZG efficiently aided in bone-related diseases therapy [33–35]. For instance, the osteoporosis model mice treated by Psoraleae Fructus displayed exceptionally less levels of OPG, RANKL, OCN and TRACP-5b proteins, and also remarkable improvement of bone loss in aspects of BV/TV, Tb.N, BMD, etc. [33].

Furthermore, years of studies have found the suppressive functions of multiple bioactive components from WSZG on BCBM progression through different ways. Osthole, one main component of Cnidii Fructus, is capable of inhibiting osteoclast differentiation and thus bone resorption in BCBM mice [34, 35]. Psoralen, a coumarin of Psoraleae Fructus, suppresses the hsa-miR-125b-5p carried by exosomes to enhance osteogenesis, which is an instruction for BCBM therapy [48, 49]. Corylifol A, a flavonoid of Psoraleae Fructus, has been verified capable of downregulating osteoclast marker levels and preventing trabecular bone loss in vivo [50]. Bakuchiol and bavachin from Psoraleae Fructus exhibit intensely bone-protection efficacies by improving bone loss [51]. Hence, these bioactive components of WSZG may be responsible for attenuating BPMN formation in BC mice.

Existed literatures have affirmed that integrins carried by exosomes play a crucial role in BC metastasis progression [31, 48]. For instance, exosomal integrin αVβ3 promoted osteoclast differentiation, which thus drove the bone metastasis of ER^+^ BC [31]. Exosomal ITGα6β4 derived from primary BC activated the Src-S100 A4 axis in lung fibroblasts to induce pre-metastatic niche formation [12]. In this study, higher expression of ITGβ3 and ITGα3 was observed in bone-tropic tumor exosomes (BO-exo), whereas WSZG pretreatment significantly decreased the expression of these two exosomal integrins. Therefore, we propose that WSZG possibly reduces the biological functions of bone-tropic exosomal integrins to attenuates BC BPMN formation. How the specific exosomal integrins trigger BPMN formation and the potential mechanism of WSZG need further investigation in future.

## Conclusion

**Fig. 8:**
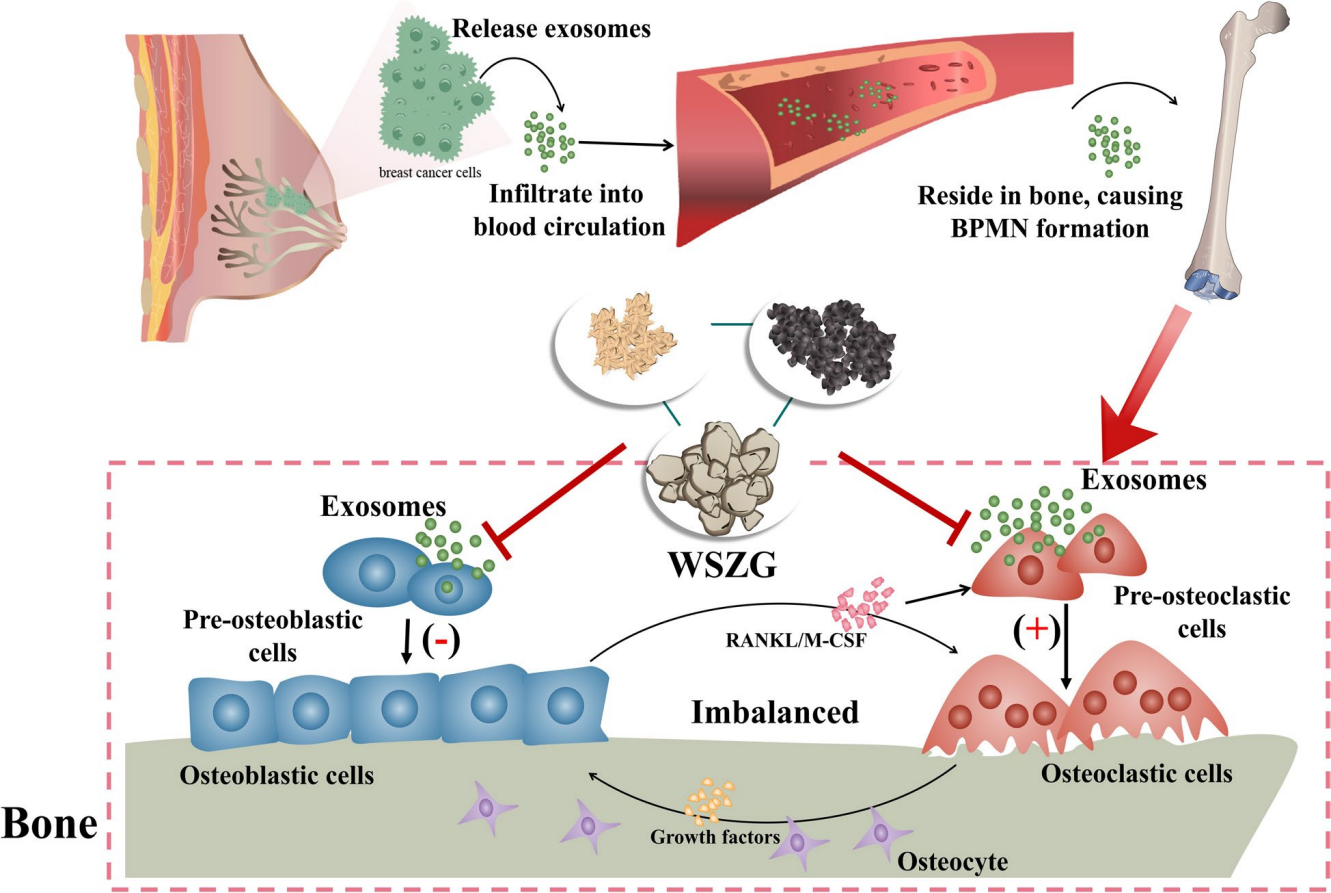
Schematic diagram of WSZG attenuating BPMN formation through repressing the absorption ability of bone precursor cells to exosomes secreted by primary breast cancer cells

To conclusion (Fig. 8), this study confirmed that the pre-education of BO-exo characterized the abnormal progression of bone microenvironment and elicited BPMN formation in primary BC mice. WSZG inhibited osteoclastic differentiation, thus improved bone destruction and restrained bone ECM protein expressional levels through targeting BO-exo. Taken together, BO-exo is regarded as a forced potential in driving BPMN emergence. And WSZG demonstrates the potential to inhibit BPMN formation in animal models and may serve as a candidate for early intervention in BCBM, thus providing a more precise insight on prevention of this disease.

## Supplementary Information

Supplementary material 1. Fig. S1 The UPLC-Q-TOF/MS chromatograms of the WSZG extract detected in positiveion modes and negativeion modes

Supplementary material 2. Fig. S2 Western blotting analyses of ACP5 and Ctsk in osteoclastic RAW 264.7 cells simultaneously exposed to WSZG and BO-exo. All data are presented as means±SEM. *p < 0.05, **p < 0.01. *ns*, no significant difference

Supplementary material 3. Fig. S3 Representative images and quantitative analysis of TRAP staining, western blot and qRT-PCR analyses of differentiated RAW 264.7 cells exposed to BO-exo that was isolated from WSZG-treated MDA-MB-231BO cells or not. All data are presented as means±SEM. **p < 0.01*. ns*, no significant difference

Supplementary material 4. Western blot analysis of ITGαV, ITGα3 and ITGβ3 in BO-exo isolated from MDA-MB-231BO cells that had been treated by WSZG or not. All data are presented as means±SEM. *p < 0.05, **p < 0.01. *ns*, no significant difference

## Data Availability

The data produced from this study are available from the corresponding author on reasonable request.

## Abbreviations

ALP: Alkaline phosphatase
BALP: Bone alkaline phosphatase
BC: Breast cancer
BCBM: Breast cancer bone metastasis
BLI: Bioluminescence imaging
BMD: Bone mineral density
BO-exo: Exosomes derived from MDA-MB-231BO cells
BPMN: Bone pre-metastasis niche
BSP: Bone sialoprotein
BV/TV: Bone volume/tissue volume
Conn-Dens.: Connectivity density
ECM: Extracellular matrix
ELISA: Enzyme linked immunosorbent assay
hBMSCs: Human bone marrow-derived mesenchymal stem cells
ICTP: Cross-linked carboxyterminal telopeptide of type I collagen
IHC: Immunohistochemistry
micro-CT: Micro computed tomography
NTA: Nanoparticle tracking analysis
OPN: Osteopontin
PEG: Polyethylene glycol
Tb.N: Trabecular number
Tb.Sp: Trabecular space
Tb.Th: Trabecular Thickness
TCM: Traditional Chinese medicine
TEM: Transmission electron microscopy
TRAP: Tartrate resistant acid phosphatase
TRACP-5b: Tartrate-resistant acid phosphatase 5b
WSZG: Wenshen Zhuanggu formula
Zole: Zoledronic acid

## References

[1] Bray F, Laversanne M, Sung H, Ferlay J, Siegel RL, Soerjomataram I, et al. Global cancer statistics 2022: GLOBOCAN estimates of incidence and mortality worldwide for 36 cancers in 185 countries. CA Cancer J Clin. 2024;74:229–63. 10.3322/caac.21834. 38572751 10.3322/caac.21834

[2] Fornetti J, Welm AL, Stewart SA. Understanding the bone in cancer metastasis. J Bone Miner Res. 2018;33:2099–113. 10.1002/jbmr.3618. 30476357 10.1002/jbmr.3618

[3] Nolan E, Kang Y, Malanchi I. Mechanisms of organ-specific metastasis of breast cancer. Cold Spring Harb Perspect Med. 2023. 10.1101/cshperspect.a041326. 36987584 10.1101/cshperspect.a041326 PMC10626265

[4] Tahara RK, Brewer TM, Theriault RL, Ueno NT. Bone metastasis of breast cancer. Adv Exp Med Biol. 2019;1152:105–29. 10.1007/978-3-030-20301-6_7. 31456182 10.1007/978-3-030-20301-6_7

[5] Yue Z, Niu X, Yuan Z, Qin Q, Jiang W, He L, et al. RSPO2 and RANKL signal through LGR4 to regulate osteoclastic premetastatic niche formation and bone metastasis. J Clin Invest. 2022. 10.1172/jci144579. 34847079 10.1172/JCI144579 PMC8759794

[6] Yuan X, Qian N, Ling S, Li Y, Sun W, Li J, et al. Breast cancer exosomes contribute to pre-metastatic niche formation and promote bone metastasis of tumor cells. Theranostics. 2021;11:1429–45. 10.7150/thno.45351. 33391543 10.7150/thno.45351 PMC7738874

[7] Kolb ADKMB. The bone extracellular matrix as an ideal milieu for cancer cell metastases. Cancers. 2019. 10.3390/cancers11071020. 31330786 10.3390/cancers11071020 PMC6678871

[8] Chen C, Huang R, Zhou J, Guo L, Xiang S. Formation of pre-metastatic bone niche in prostate cancer and regulation of traditional chinese medicine. Front Pharmacol. 2022;13: 897942. 10.3389/fphar.2022.897942. 36059977 10.3389/fphar.2022.897942 PMC9428453

[9] Hofbauer LC, Bozec A, Rauner M, Jakob F, Perner S, Pantel K. Novel approaches to target the microenvironment of bone metastasis. Nat Rev Clin Oncol. 2021;18:488–505. 10.1038/s41571-021-00499-9. 33875860 10.1038/s41571-021-00499-9

[10] Tiedemann K, Sadvakassova G, Mikolajewicz N, Juhas M, Sabirova Z, Tabariès S, et al. Exosomal release of L-plastin by breast cancer cells facilitates metastatic bone osteolysis. Transl Oncol. 2019;12:462–74. 10.1016/j.tranon.2018.11.014. 30583289 10.1016/j.tranon.2018.11.014 PMC6305809

[11] Simpson RJ, Lim JW, Moritz RL, Mathivanan S. Exosomes: proteomic insights and diagnostic potential. Expert Rev Proteom. 2009;6:267–83. 10.1586/epr.09.17. 10.1586/epr.09.17 19489699

[12] Hoshino A, Costa-Silva B, Shen TL, Rodrigues G, Hashimoto A, Tesic Mark M, et al. Tumour exosome integrins determine organotropic metastasis. Nature. 2015;527:329–35. 10.1038/nature15756. 26524530 10.1038/nature15756 PMC4788391

[13] Huang Q, Wang J, Ning H, Liu W, Han X. Integrin β1 in breast cancer: mechanisms of progression and therapy. Breast Cancer. 2025;32:43–59. 10.1007/s12282-024-01635-w. 39343856 10.1007/s12282-024-01635-w

[14] Sadu L, Krishnan RH, Akshaya RL, Das UR, Satishkumar S, Selvamurugan N. Exosomes in bone remodeling and breast cancer bone metastasis. Prog Biophys Mol Biol. 2022;175:120–30. 10.1016/j.pbiomolbio.2022.09.008. 36155749 10.1016/j.pbiomolbio.2022.09.008

[15] Bai N, Wang Q, Zhang C, Wang J. Triterpenoid saponin from *Ardisia**gigantifolia**Stapf* inhibits cancer cell metastasis behavior of TNBC via suppressing EMT and stemness through ITGB4/FAK signals. Clinical Tradit Med Pharmacol. 2024;5: 200182. 10.1016/j.ctmp.2024.200182.

[16] Wang Y, Li JW, Qin YN, Sun CP, Chen JJ, Ruan YY, et al. Clinical observation on the effect of Chinese medicine-"TCM formula" intervention on recurrence and metastasis of triple negative breast cancer. Complement Ther Med. 2020;52: 102456. 10.1016/j.ctim.2020.102456. 32951717 10.1016/j.ctim.2020.102456

[17] Huang X, Wang J, Lin W, Zhang N, Du J, Long Z, et al. Kanglaite injection plus platinum-based chemotherapy for stage III/IV non-small cell lung cancer: a meta-analysis of 27 RCTs. Phytomedicine. 2020;67: 153154. 10.1016/j.phymed.2019.153154. 31926475 10.1016/j.phymed.2019.153154

[18] Sun L, Yan Y, Chen D, Yang Y. Quxie capsule modulating gut microbiome and its association with T cell regulation in patients with metastatic colorectal cancer: result from a randomized controlled clinical trial. Integr Cancer Ther. 2020;19:1534735420969820. 10.1177/1534735420969820. 33243018 10.1177/1534735420969820 PMC7876934

[19] Chen Q, Shu C, Laurence AD, Chen Y, Peng BG, Zhen ZJ, et al. Effect of Huaier granule on recurrence after curative resection of HCC: a multicentre, randomised clinical trial. Gut. 2018;67:2006–16. 10.1136/gutjnl-2018-315983. 29802174 10.1136/gutjnl-2018-315983

[20] Chen J. Clinical Study on the Treatment of “Wenshen Zhuanggu” Method for Bone Metastatic Pain in Breast Cancer (Master’s Dissertation). Shanghai: Shanghai University of Traditional Chinese Medicine; 2018.

[21] Li JJ, Chen WL, Wang JY, Hu QW, Sun ZP, Zhang S, et al. Wenshen Zhuanggu formula effectively suppresses breast cancer bone metastases in a mouse Xenograft model. Acta Pharmacol Sin. 2017;38:1369–80. 10.1038/aps.2017.13. 28414206 10.1038/aps.2017.13 PMC5630667

[22] Han XH, Wang CL, Xie Y, Ma J, Zhang XH, Hu QW, et al. Anti-metastatic effect and mechanisms of Wenshen Zhuanggu formula in human breast cancer cells. J Ethnopharmacol. 2015;162:39–46. 10.1016/j.jep.2014.12.036. 25554638 10.1016/j.jep.2014.12.036

[23] Ma J, Li J, Wang Y, Chen W, Zheng P, Chen Y, et al. WSZG inhibits BMSC-induced EMT and bone metastasis in breast cancer by regulating TGF-β1/Smads signaling. Biomed Pharmacother. 2020;121: 109617. 10.1016/j.biopha.2019.109617. 31810139 10.1016/j.biopha.2019.109617

[24] Rider MA, Hurwitz SN, Meckes DG. ExtraPEG: a polyethylene glycol-based method for enrichment of extracellular vesicles. Sci Rep. 2016;6:23978. 10.1038/srep23978. 27068479 10.1038/srep23978 PMC4828635

[25] Zhang Y, Zuo B, Yu Z, Zhao K, Zhang Y, He K, et al. Complete remission of tumors in mice with neoantigen-painted exosomes and anti-PD-1 therapy. Mol Ther. 2023;31:3579–93. 10.1016/j.ymthe.2023.10.021. 37919900 10.1016/j.ymthe.2023.10.021 PMC10727972

[26] Liu X, Chen M, Luo J, Zhao H, Zhou X, Gu Q, et al. Immunopolarization-regulated 3D printed-electrospun fibrous scaffolds for bone regeneration. Biomaterials. 2021;276: 121037. 10.1016/j.biomaterials.2021.121037. 34325336 10.1016/j.biomaterials.2021.121037

[27] Chen S, Ye X, Yu X, Xu Q, Pan K, Lu S, et al. Co-culture with periodontal ligament stem cells enhanced osteoblastic differentiation of MC3T3-E1 cells and osteoclastic differentiation of RAW264.7 cells. Int J Clin Exp Pathol. 2015;8:14596–607. 26823783 PMC4713569

[28] Kong L, Ma R, Cao Y, Smith W, Liu Y, Yang X, et al. Cell cytoskeleton and proliferation study for the RANKL-induced RAW264.7 differentiation. J Cell Mol Med. 2021;25:4649–57. 10.1111/jcmm.16390. 33742541 10.1111/jcmm.16390 PMC8107080

[29] Ma C, Geng B, Zhang X, Li R, Yang X, Xia Y. Fluid shear stress suppresses osteoclast Differentiation in RAW264 7 cells through extracellular signal-regulated kinase 5 (ERK5) signaling pathway. Med Sci Monit. 2020;26:e918370. 10.2659/msm.918370. 31914120 10.12659/MSM.918370 PMC6977602

[30] Dai R, Liu M, Xiang X, Xi Z, Xu H. Osteoblasts and osteoclasts: an important switch of tumour cell dormancy during bone metastasis. J Exp Clin Cancer Res. 2022;41:316. 10.1186/s13046-022-02520-0. 36307871 10.1186/s13046-022-02520-0 PMC9615353

[31] Wu K, Feng J, Lyu F, Xing F, Sharma S, Liu Y, et al. Exosomal miR-19a and IBSP cooperate to induce osteolytic bone metastasis of estrogen receptor-positive breast cancer. Nat Commun. 2021;12:5196. 10.1038/s41467-021-25473-y. 34465793 10.1038/s41467-021-25473-y PMC8408156

[32] Chen B, Sang Y, Song X, Zhang D, Wang L, Zhao W, et al. Exosomal miR-500a-5p derived from cancer-associated fibroblasts promotes breast cancer cell proliferation and metastasis through targeting USP28. Theranostics. 2021;11:3932–47. 10.7150/thno.53412. 33664871 10.7150/thno.53412 PMC7914354

[33] Lin Z, Zheng J, Chen J, Chen M, Dong S. antiosteoporosis effect and possible mechanisms of the ingredients of fructus psoraleae in animal models of osteoporosis: a preclinical systematic review and meta-analysis. Oxid Med Cell Longev. 2021;2021:2098820. 10.1155/2021/2098820. 34868453 10.1155/2021/2098820 PMC8635882

[34] Ma Y, Wang L, Zheng S, Xu J, Pan Y, Tu P, et al. Osthole inhibits osteoclasts formation and bone resorption by regulating NF-κB signaling and NFATc1 activations stimulated by RANKL. J Cell Biochem. 2019;120:16052–61. 10.1002/jcb.28886. 31081953 10.1002/jcb.28886

[35] Wu C, Sun Z, Guo B, Ye Y, Han X, Qin Y, et al. Osthole inhibits bone metastasis of breast cancer. Oncotarget. 2017;8:58480–93. 10.1632/oncotarget.17024. 28938572 10.18632/oncotarget.17024 PMC5601668

[36] Kruger TE, Miller AH, Godwin AK, Wang J. Bone sialoprotein and osteopontin in bone metastasis of osteotropic cancers. Crit Rev Oncol Hematol. 2014;89:330–41. 10.1016/j.critrevonc.2013.08.013. 24071501 10.1016/j.critrevonc.2013.08.013 PMC3946954

[37] Satcher RLXHZ. Evolving cancer-niche interactions and therapeutic targets during bone metastasis. Nat Rev Cancer. 2022;22:85–101. 10.1038/s41568-021-00406-5. 34611349 10.1038/s41568-021-00406-5 PMC10281546

[38] Yu T, Wang C, Xie M, Zhu C, Shu Y, Tang J, et al. Heterogeneity of CTC contributes to the organotropism of breast cancer. Biomed Pharmacother. 2021;137: 111314. 10.1016/j.biopha.2021.111314. 33581649 10.1016/j.biopha.2021.111314

[39] Liu YXCao. Characteristics and significance of the pre-metastatic niche. Cancer Cell. 2016;30:668–81. 10.1016/j.ccell.2016.09.011. 27846389 10.1016/j.ccell.2016.09.011

[40] Maji S, Chaudhary P, Akopova I, Nguyen PM, Hare RJ, Gryczynski I, et al. Exosomal annexin II promotes angiogenesis and breast cancer metastasis. Mol Cancer Res. 2017;15:93–105. 10.1158/1541-7786.Mcr-16-0163. 27760843 10.1158/1541-7786.MCR-16-0163 PMC5215956

[41] Shao Y, Chen T, Zheng X, Yang S, Xu K, Chen X, et al. Colorectal cancer-derived small extracellular vesicles establish an inflammatory premetastatic niche in liver metastasis. Carcinogenesis. 2018;39:1368–79. 10.1093/carcin/bgy115. 30184100 10.1093/carcin/bgy115

[42] Taverna S, Pucci M, Giallombardo M, Di Bella MA, Santarpia M, Reclusa P, et al. Amphiregulin contained in NSCLC-exosomes induces osteoclast differentiation through the activation of EGFR pathway. Sci Rep. 2017;7:3170. 10.1038/s41598-017-03460-y. 28600504 10.1038/s41598-017-03460-y PMC5466625

[43] Zhou CF, Ma J, Huang L, Yi HY, Zhang YM, Wu XG, et al. Cervical squamous cell carcinoma-secreted exosomal miR-221-3p promotes lymphangiogenesis and lymphatic metastasis by targeting VASH1. Oncogene. 2019;38:1256–68. 10.1038/s41388-018-0511-x. 30254211 10.1038/s41388-018-0511-x PMC6363643

[44] Dai J, Escara-Wilke J, Keller JM, Jung Y, Taichman RS, Pienta KJ, et al. Primary prostate cancer educates bone stroma through exosomal pyruvate kinase M2 to promote bone metastasis. J Exp Med. 2019;216:2883–99. 10.1084/jem.20190158. 31548301 10.1084/jem.20190158 PMC6888980

[45] Zhou J, Song Q, Li H, Han Y, Pu Y, Li L, et al. Targeting circ-0034880-enriched tumor extracellular vesicles to impede SPP1(high)CD206(+) pro-tumor macrophages mediated pre-metastatic niche formation in colorectal cancer liver metastasis. Mol Cancer. 2024;23:168. 10.1186/s12943-024-02086-9. 39164758 10.1186/s12943-024-02086-9 PMC11334400

[46] Xu L, Faruqu FN, Liam-Or R, Abu Abed O, Li D, Venner K, et al. Design of experiment (DoE)-driven in vitro and in vivo uptake studies of exosomes for pancreatic cancer delivery enabled by copper-free click chemistry-based labelling. J Extracell Vesicles. 2020;9:1779458. 10.1080/20013078.2020.1779458. 32944169 10.1080/20013078.2020.1779458 PMC7480572

[47] Morad G, Carman CV, Hagedorn EJ, Perlin JR, Zon LI, Mustafaoglu N, et al. Tumor-derived extracellular vesicles breach the intact blood-brain barrier via transcytosis. ACS Nano. 2019;13:13853–65. 10.1021/acsnano.9b04397. 31479239 10.1021/acsnano.9b04397 PMC7169949

[48] Carter RZ, Micocci KC, Natoli A, Redvers RP, Paquet-Fifield S, Martin AC, et al. Tumour but not stromal expression of β3 integrin is essential, and is required early, for spontaneous dissemination of bone-metastatic breast cancer. J Pathol. 2015;235:760–72. 10.1002/path.4490. 25430721 10.1002/path.4490

[49] Yu J, Wu X, Zhang W, Chu F, Zhang Q, Gao M, et al. Effect of psoralen on the regulation of osteogenic differentiation induced by periodontal stem cell-derived exosomes. Hum Cell. 2023;36(4):1389–402. 10.1007/s13577-023-00918-2 10.1007/s13577-023-00918-2 PMC10284944 37269415

[50] Xu Y, Song D, Lin X, Peng H, Su Y, Liang J, et al. Corylifol A protects against ovariectomized-induced bone loss and attenuates RANKL-induced osteoclastogenesis via ROS reduction, ERK inhibition, and NFATc1 activation. Free Radic Biol Med. 2023;196:121–32. 10.1016/j.freeradbiomed.2023.01.017 10.1016/j.freeradbiomed.2023.01.017 36649902

[51] Weng Z-B, Gao Q-Q, Wang F, Zhao G-H, Yin F-Z, Cai B-C, et al. Positive skeletal effect of two ingredients of Psoralea corylifolia L. on estrogen deficiency-induced osteoporosis and the possible mechanisms of action. Mol Cell Endocrinol. 2015;417:103–13. 10.1016/j.mce.2015.09.025 10.1016/j.mce.2015.09.025 26419930

