# Supplementary figures and images for: Wenshen Zhuanggu formula inhibits tumor-exosomes induced bone pre-metastasis niche formation in primary breast cancer mice
Source: Chin Med. 2025 Jun 16;20:88. doi: 10.1186/s13020-025-01136-8 (PMC12168286; doi:10.1186/s13020-025-01136-8)

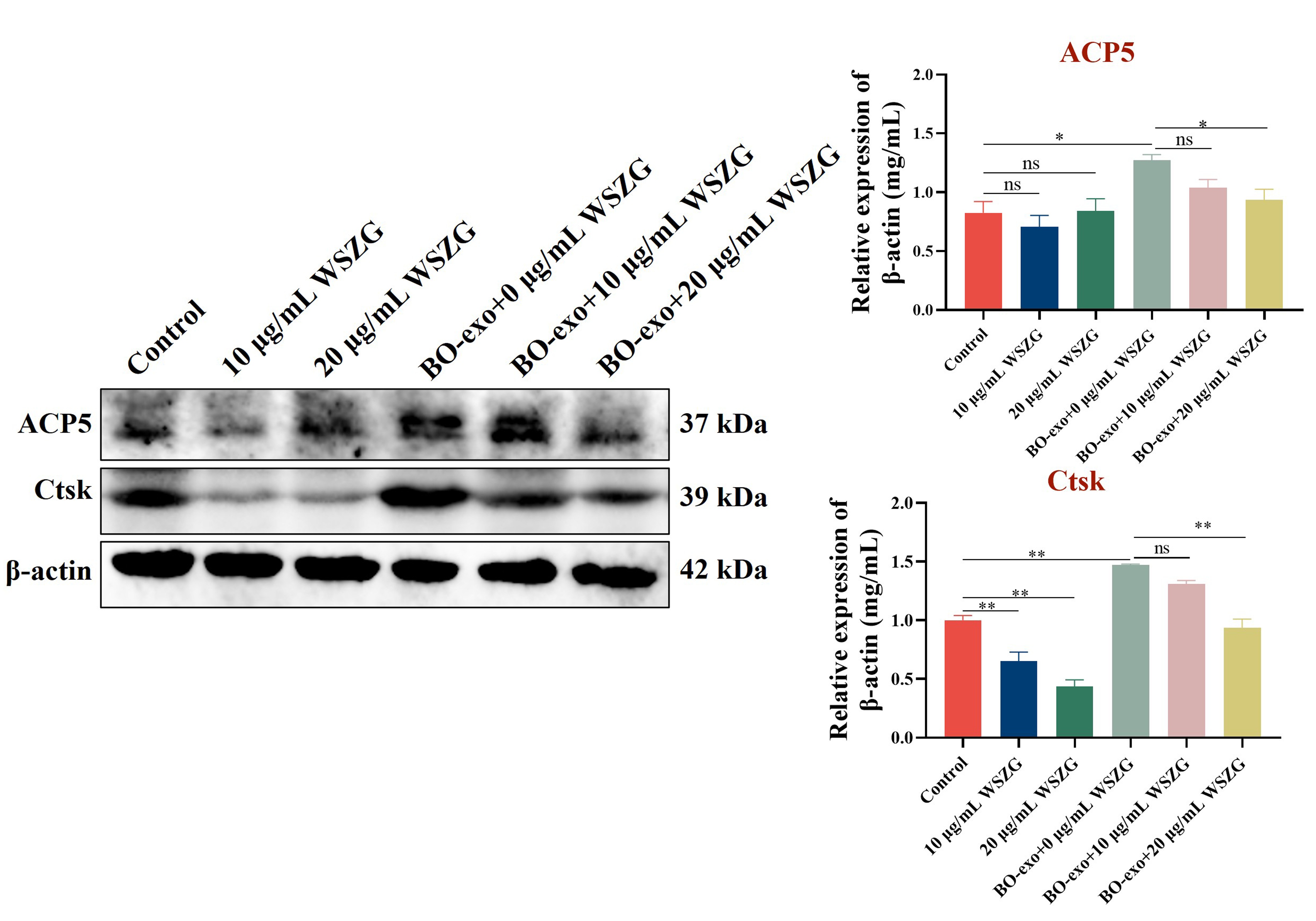

Supplement: Supplementary file 2 — Supplementary material 2. Fig. S2 Western blotting analyses of ACP5 and Ctsk in osteoclastic RAW 264.7 cells simultaneously exposed to WSZG and BO-exo. All data are presented as means±SEM. *p < 0.05, **p < 0.01. ns, no significant difference [file 13020_2025_1136_MOESM2_ESM.tif]

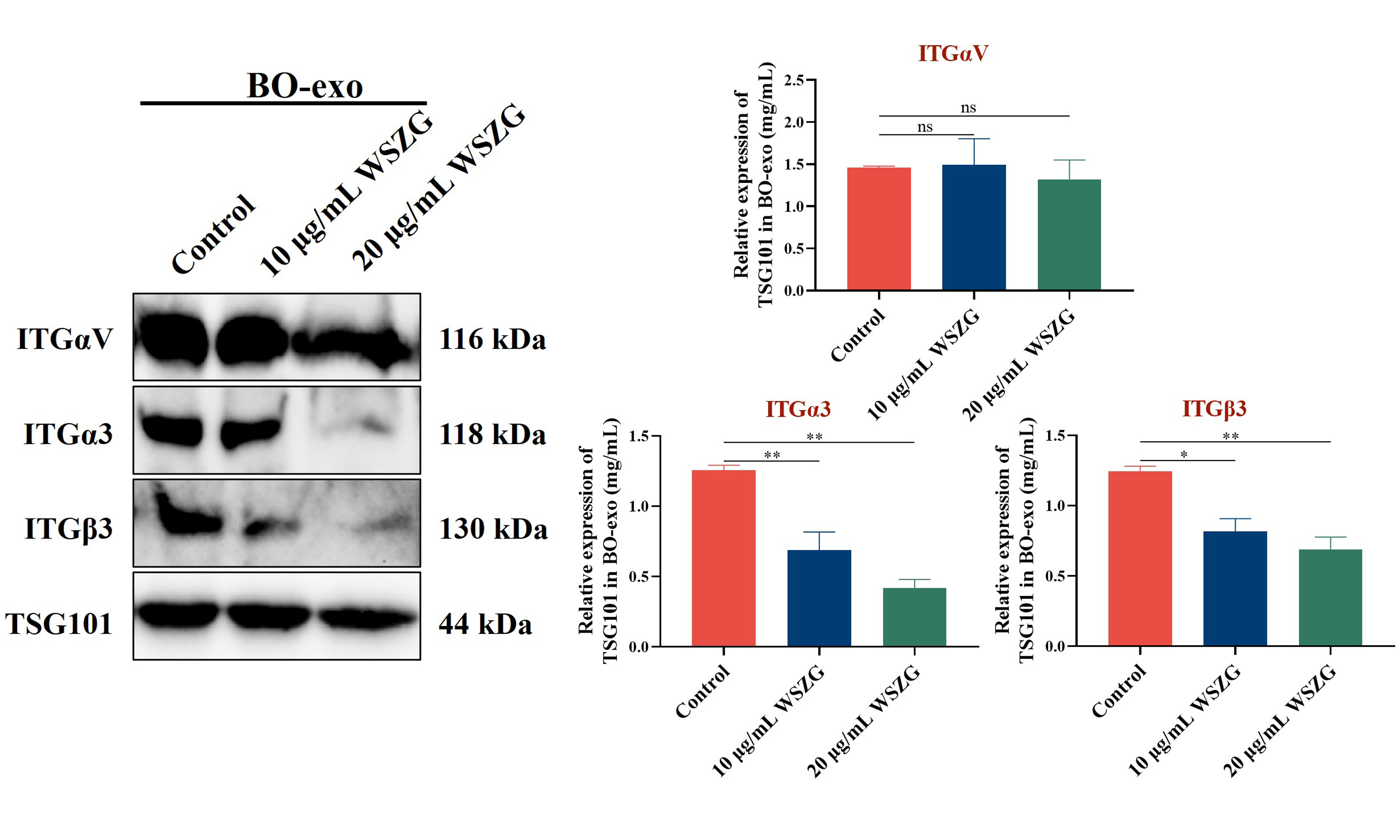

Supplement: Supplementary file 4 — Supplementary material 4. Western blot analysis of ITGαV, ITGα3 and ITGβ3 in BO-exo isolated from MDA-MB-231BO cells that had been treated by WSZG or not. All data are presented as means±SEM. *p < 0.05, **p < 0.01. ns, no significant difference [file 13020_2025_1136_MOESM4_ESM.tif]
